# Node Deployment Algorithm Based on Connected Tree for Underwater Sensor Networks

**DOI:** 10.3390/s150716763

**Published:** 2015-07-10

**Authors:** Peng Jiang, Xingmin Wang, Lurong Jiang

**Affiliations:** 1 Key Lab for IOT and Information Fusion Technology of Zhejiang, Hangzhou Dianzi University, Hangzhou 310018, China; E-Mail: wangxm@hdu.edu.cn; 2 School of Information Science and Technology, Zhejiang Sci-Tech University, Hangzhou 310018, China; E-Mail: jianglurong@zstu.edu.cn

**Keywords:** underwater sensor network deployment, node depth adjustment, 3D coverage, connected tree

## Abstract

Designing an efficient deployment method to guarantee optimal monitoring quality is one of the key topics in underwater sensor networks. At present, a realistic approach of deployment involves adjusting the depths of nodes in water. One of the typical algorithms used in such process is the self-deployment depth adjustment algorithm (SDDA). This algorithm mainly focuses on maximizing network coverage by constantly adjusting node depths to reduce coverage overlaps between two neighboring nodes, and thus, achieves good performance. However, the connectivity performance of SDDA is irresolute. In this paper, we propose a depth adjustment algorithm based on connected tree (CTDA). In CTDA, the sink node is used as the first root node to start building a connected tree. Finally, the network can be organized as a forest to maintain network connectivity. Coverage overlaps between the parent node and the child node are then reduced within each sub-tree to optimize coverage. The hierarchical strategy is used to adjust the distance between the parent node and the child node to reduce node movement. Furthermore, the silent mode is adopted to reduce communication cost. Simulations show that compared with SDDA, CTDA can achieve high connectivity with various communication ranges and different numbers of nodes. Moreover, it can realize coverage as high as that of SDDA with various sensing ranges and numbers of nodes but with less energy consumption. Simulations under sparse environments show that the connectivity and energy consumption performances of CTDA are considerably better than those of SDDA. Meanwhile, the connectivity and coverage performances of CTDA are close to those depth adjustment algorithms base on connected dominating set (CDA), which is an algorithm similar to CTDA. However, the energy consumption of CTDA is less than that of CDA, particularly in sparse underwater environments.

## 1. Introduction

Underwater wireless sensor networks (UWSNs) are underwater monitoring systems that comprise nodes with acoustic communication and computational capability [1]. Typical applications of UWSNs include, but are not limited to, underwater tactical surveillance, resource survey, and environmental monitoring [2,3,4,5]. UWSNs mainly involve aspects of underwater communication technology, network protocol design, node deployment, underwater simulation system design, underwater localization and target tracking, time synchronization, reliable data transmission and storage management, power management, and underwater security [6,7,8,9,10,11,12]. Deployment is one of the most fundamental issues in UWSNs because it does not only promote underwater monitoring quality but also reduce network construction cost, among other aspects [13]. An appropriate node deployment scheme can also be a good foundation for subsequent network communication protocol designs and operations. Thus, establishing methods to deploy nodes is a key issue that should be urgently addressed.

Node deployment in UWSNs exhibits features that are different from those of node deployment in terrestrial wireless sensor networks. For example, nodes are generally deployed in 3D space in the former; other differences include the complex underwater environment, sparse node distribution, and the influence of water flow on node movement [14,15,16]. Underwater node deployment algorithms can be grouped into static and dynamic deployments according to the movement capability of nodes [17,18]. In static deployment, node position requires pre-calculation, and nodes should be placed manually on predetermined locations [16]. However, static deployment is inappropriate in many UWSN cases, such as underwater resource exploration and tactical surveillance. Moreover, when the volume of the monitored area increases, such manual deployment will require considerable additional manpower and material resources. In dynamic deployment, nodes can move and can be repositioned dynamically when network topology changes. Based on the movement capability of nodes, dynamic deployment algorithms can be divided into limited deployment and free deployment algorithms. The former assumes that nodes can only move in a certain direction, which is typically vertical; its correlated algorithms are called depth adjustment algorithms. By contrast, a free deployment algorithm assumes that nodes can move freely in water and can reach any position. In UWSNs, free deployment, such as network topology adjustment that uses an autonomous underwater vehicle, leads to high energy and resource costs [19,20]. Thus, limited deployment is more practical than free deployment in UWSNs. Some researchers have added a depth adjustment module to the limited deployment of underwater nodes, and thus, realized vertical movement in underwater environments.

In this paper, we propose a distributed deployment algorithm by adjusting node positions vertically and building connected trees to maintain network connectivity. This algorithm, which is called a depth adjustment algorithm based on connected tree (CTDA), minimizes coverage overlap between the root and child nodes in a sub-tree to improve network coverage. CTDA can maximize network coverage while reducing energy consumption under the premise of ensuring network connectivity. CTDA assumes that nodes in UWSNs are divided into two categories, *i.e.*, sink and sensor nodes, and that all nodes can only be moved vertically. After the SINK node and sensor nodes are randomly deployed on the water surface, the sink node, which functions as the root node, starts to build a connected tree. Finally, the network is organized as a forest that consists of many connected sub-trees. The depth of the leaf node of each parent node is adjusted based on the coverage overlap between two nodes to minimize overlaps and improve coverage. The distance between the root and child nodes is adjusted hierarchically to reduce node moving distance, and the silent mode is adopted to reduce communication energy. CTDA is compared with the random uniform distributed algorithm (RAND) and the self-deployment depth adjustment algorithm (SDDA) [21] in terms of network coverage, connectivity, and deployment energy consumption under different node sensing ranges, communication ranges, and numbers of nodes. The simulation results show that CTDA always maintains high network connectivity under different communication ranges and numbers of nodes, while achieving high network coverage similar to that of SDDA under different sensing ranges and numbers of nodes but with less deployment energy consumption. The simulations under a sparse network show that the connectivity and deployment energy consumption performances of CTDA are considerably better than those of SDDA. Meanwhile, the connectivity and coverage performances of CTDA are close to those depth adjustment algorithms base on connected dominating set (CDA) [22], which is an algorithm similar to CTDA. However, the energy consumption of CTDA is less than that of CDA, particularly in sparsely distributed environments.

This paper is organized as follows. Section 2 summarizes related works. Section 3 describes the system model and the assumptions considered in this study. Section 4 presents relevant definitions and the details of CTDA. Section 5 analyzes the complexity of CTDA. Section 6 discusses the performance study and provides a detailed analysis of its result. Finally, Section 7 concludes the paper.

## 2. Related Works

Numerous works have focused on node deployment in UWSNs.

Ravelomanana [23] analyzed the implications of sensing and communication ranges on coverage, connectivity, and network diameter, and obtained constraint relations and theoretical limits among number of nodes, node sensing radius, and communication radius. These results were based on probability, and this literature focused on networks with nodes that were randomly and uniformly distributed.

Pompili *et al.* [24] proposed a bottom-grid algorithm based on triangular deployment in the seabed by adjusting the depth of nodes to achieve 3D coverage. This algorithm requires the global information of nodes and belongs to the centralized approach. This study did not refer to the problem on depth adjustment through the sink node and did not discuss the connectivity problem.

Alam and Hass [25] investigated the problem of achieving maximum coverage in a given 3D space with the least number of sensor nodes. They were inspired by the idea of the 3D Voronoi polyhedron filling space. This study compared the volume quotient of a truncated octahedron, a cube, a regular hexagonal prism, and a rhombic dodecahedron with their respective external sphere. The authors proved that the required number of nodes was minimal when the truncated octahedron mode was adopted, and they concluded that connectivity was ensured if the communication range of the nodes was at least 1.79 times of the sensing range. This study has also provided an equation for calculating node position, *i.e.*, Equation (1), where (*cx*, *cy*, *cz*) is the center point of the *x*, *y*, *z* coordinate system; and *u_1_*, *v_1_*, *w_1_* are the positive integers. This algorithm is also a centralized algorithm, and sensor nodes are placed manually on predetermined locations.

(1) $$\{\begin{array}{ll} x_{i}= & cx+(2u_{1}+w_{1})\frac{2s}{\sqrt{5}}\\ y_{i}= & cy+(2v_{1}+w_{1})\frac{2s}{\sqrt{5}}\\ z_{i}= & cz+w_{1}\frac{2s}{\sqrt{5}} \end{array}$$

Akkaya *et al.* [21] presented SDDA, whose basic idea was to adjust the depth of sensor nodes after their initial deployment to reduce coverage overlaps between two neighboring nodes. The depth adjustment process would continue until sensor coverage could no longer be improved. This algorithm exhibited good coverage performance, but network connectivity was ignored at the initial stage. Although this algorithm could continue node depth adjustment to improve network connectivity, energy consumption would increase and coverage might decrease. In addition, network topology in a sparse network might degenerate from 3D to 2D.

Senel *et al.* [22] also proposed a node depth adjustment algorithm. This algorithm initially constructs the connected backbone [26], and then uses the dominating node on the backbone to calculate the depth of the dominated node iteratively. In this algorithm, nodes are required to communicate mutely with other nodes. Moreover, the number of iterations increases with the number of nodes, and the amount of calculation considerably rises.

Zen *et al.* [27] proposed a node-moving algorithm that considered the influence of water flow. This algorithm employs water force to save energy and investigates the problem of compensating for local area coverage after the failure of nodes.

Xia *et al.* [28] established joint optimization objectives for underwater sensor network coverage and connectivity following a strict theoretical basis, and proposed a corresponding moving algorithm. This algorithm enables networks to improve coverage and connectivity. However, it requires nodes to have good movement capability and accurate underwater positioning.

## 3. System Model and Assumptions

Sink and sensor nodes are randomly spread in a monitored area, floating on the water surface with buoys. First, the locations of the sensor and sink nodes are determined by running a localization algorithm or by using Global Positioning Satellite devices. The positions of the sensor nodes are then adjusted underwater by moving them to form a 3D coverage network. However, the mobility of sensor nodes is limited with the current technology. Available underwater sensor nodes can only move vertically because horizontal movement is impossible, except with the effect of water flow.

Notably, adjusting the depth of an underwater sensor is possible by using various current technologies. For example, Bokser *et al.* [29] introduced an underwater sensor node that used a cylinder to drown or force water into the node to control its depth. Detweiler *et al.* [30] described a depth adjustment device that could control node depth by adjusting motor cable length. Jaffe *et al.* [31] described a spherical node that could be submerged to a predetermined depth.

**Figure 1:**
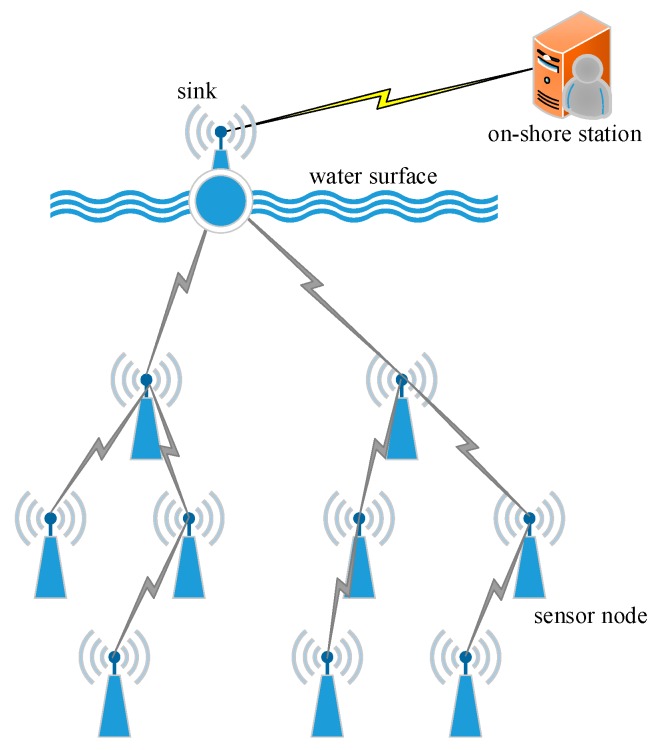
System model.

A typical UWSNs architecture is depicted in Figure 1. In this model, sensor nodes communicate with one another through acoustic channels and maintain connectivity with the sink node via one-hop or multi-hop paths. The node is fixed at its position by an anchor. Meanwhile, the following assumptions are made:

(1) A node adopts the Boolean perceptual model, the sensing range *R_s_* represents its ability to monitor the surroundings. If the sensing range of any node is *R_s_*, then the sensing field of the node is spherical, wherein the node is the center.

(2) The communication range *R_c_* of a node represents its theoretically max communicating range with other nodes, the broadcast range *R_b_* of a node represents its real communicating range with other nodes. A node can adjust its broadcast range *R_b_* by adjusting its transmission power.

(3) All nodes are homogeneous, they have the same *R_s_* and *R_c_* and the data integration capabilities, and each of them has a unique identification number (ID). However, they may have different *R_b_*.

## 4. Related Definitions and Algorithm Description

This section presents the detailed description of CTDA. Section 4.1 mainly introduces the relevant definitions involved in CTDA, Section 4.2 gives the description and analysis about the problem investigated in this paper, and Section 4.3 provides a detailed description about the concrete steps of CTDA.

### 4.1. Related Definitions

#### 4.1.1. Coverage

Assuming that the Euclidean distance between a sensor node *s_i_*(*x_i_*, *y_i_*, *z_i_*) and an arbitrary point *p*(*x*, *y*, *z*) in a 3D space is *d*(*s_i_*, *p*). The probability that point *p* is covered by sensor node *s_i_* is

(2) $$C_{p}(s_{i})=\{\begin{array}{c} 1,\;d(s_{i},p)<R_{s}\\ 0,\;d(s_{i},p)\geq R_{s} \end{array}$$

where $d(s_{i},p)=\sqrt{{\left(x-x_{i}\right)}^{2}+{\left(y-y_{i}\right)}^{2}+{\left(z-z_{i}\right)}^{2}}$, *x*, *y*, *z* are the coordinates of point *p*, and *x_i_*, *y_i_*, *z_i_* are the coordinates of sensor *s_i_*.

As shown in Equation (2), if the Euclidean distance between sensor node *s_i_* and point *p* is less than the sensing radius, then point *p* can be covered; otherwise, point *p* cannot be covered. Assuming that the network includes k nodes *s_1_*, *s_2_*, *...*, *s_k_*, and the set which consists of these nodes is called A, the respective coverage of point *p* is *c_p_*(*s_1_*), *c_p_*(*s_2_*), *...*, *c_p_*(*s_k_*), and the detection probability of point *p* is

(3) $$c_{p}(A)=1-\prod_{i=1}^{k}\left(1-c_{p}(s_{i})\right)$$

Point *p* is called the probe point. If *n* probe points exist, then the mean value of the detection probability of these probe points can be regarded as the overall network coverage, as follows:

(4) $$C(A)=\frac{1}{n}\sum_{i=1}^{n}c_{i}(A)$$

Notably, *Cp*(*A*) is the statistical value of the actual environment. When *n* is sufficiently large, the statistical value is infinitely close to the theoretical value.

#### 4.1.2. Coverage Contribution

The coverage contribution of a node is defined as the area volume covered only by a node. The expected coverage contribution of the network coverage is defined as follows:

(5) $$\text{exp}ect\_\text{cov}erage=\min\{V_{space}/n_{node},(4/3)\pi Rs^{3}\}$$

where *V_space_* is the volume of water to be monitored and *n_node_* is the total number of nodes in the network.

#### 4.1.3. Connectivity

The premise of collaborative work is to ensure connectivity in the entire network. In this case, network connectivity is defined as the ratio of the number of nodes linked to the sink node to the total number of nodes.

### 4.2. Problem Definition

Initially, sink and sensor nodes are randomly and uniformly distributed on the water surface. Afterward, the nodes descend to different water depths according to the node deployment strategy used to cover the monitoring area. During adjustment, the distance among nodes increases, which may disconnect originally interconnected nodes, and thus, decrease network connectivity. The main objective is to maximize network coverage under the premise of maintaining initial network connectivity. Each node strives to achieve the desired coverage network. Thus, in this study, the problem is defined as follows: given *N* sensor nodes that are randomly and uniformly deployed on the water surface, how to design an algorithm that maximizes network coverage under the premise of maintaining network connectivity while minimizing energy consumption.

Several methods are available to solve this problem. One of these methods is provided in [25], in which each node is allowed to move to its optimal pre-calculated location. However, this algorithm exhibits two problems. First, it requires sensor nodes to pre-calculate the optimum position. Second, sensor nodes can only be moved vertically, and their optimum position may not be reached unless the nodes are manually deployed. The second solution will be to allow the nodes to select random depths. However, this solution may not provide the desired coverage and connectivity, although it is simple and does not cause any communication overhead during deployment. The third solution is provided in [21]. First, the network begins to cluster, and then, the selected cluster head assigns a group number to each node based on the sensing overlapping region between two neighboring nodes. The group number determines node depth. After the first round of depth adjustments, the depth of the nodes continues to be adjusted further, which reduces coverage overlap or improves connectivity. Although this method can achieve high network coverage, certain problems still occur. First, network connectivity performs poorly when *R_c_* is less than 2 × *R_c_* or the depth of the monitored area is considerably larger than *R_c_*. To improve connectivity, node depth should be continuously adjusted, which increases energy consumption and decreases network coverage. Second, as the distribution of nodes becomes increasingly sparse, fewer nodes will exhibit coverage overlapping and an increasing number of nodes are assigned with the same number. Hence, node distribution becomes concentrated, and network topology is gradually reduced from 3D to 2D. Furthermore, this algorithm adopts the active communication mode, which results in high communication energy consumption. The fourth solution uses the algorithm proposed in [25], which initially constructs the connected backbone and then uses the dominating node on the backbone to calculate the depth of the dominated node of the algorithm iteratively. This algorithm considers both connectivity and coverage; however, nodes are required to communicate with other nodes while building the connected backbone and during depth calculation. Moreover, the number of iterations and the amount of calculation significantly increase with the number of nodes.

A depth adjustment algorithm (*i.e.*, CTDA) is proposed in this study by building connected trees to maintain network connectivity and minimizing coverage overlap between the root and child nodes in a sub-tree to improve network coverage. Simultaneously, a hierarchical strategy is introduced to adjust the distance between the root and child nodes to reduce the travel distance of the node. The silent mode is adopted to reduce communication energy. The details of the proposed algorithm are discussed in the following section.

### 4.3. Algorithm Description

Initially, the sink node begins to broadcast inquiry packets as the root node and the first parent node of the network. The sink node then selects nodes within the range of *R_b_* with a state flag of 0 as leaf nodes to construct the connected tree. Meanwhile, the root node selects the next root node and constructs the next connected tree through this node. Finally, the network can be organized as a forest.

Each root node follows this principle: if the coverage overlap between two nodes is the maximum, then the overlap between these nodes should be the smallest after adjustment is performed under the premise of maintaining connectivity. Therefore, the root node first regards itself as the first parent node, and then selects the nearest leaf node with a status flag of 0 away from itself as the child node in each round. Meanwhile, in each round, *R_b_* can be used as the weight value to adjust the distance between the parent and child nodes. The method to determine the value of *R_b_* is as follows:

(1) When the ratio of *R_c_/R_s_* is less than 2, *R_b_* is equal to *R_c_*.
(2) When the ratio of *R_c_/R_s_* is greater than 2, if the distance between the parent and child node is equal to 2 × *R_s_*, the coverage overlap between these two nodes is zero. Hence, *R_b_* is equal to 2 × *R_s_*.
(3) When the ratio of *R_c_/ R_s_* is greater than 2, if nodes are sparsely distributed, the child node may not exist within the range of 2 × *R_s_* away from the parent node. Therefore, a gradual adjustment mechanism is introduced to adjust the value of *R_b_*. *R_b_* is progressively increased until the maximum broadcast radius (*i.e.*, *R_c_*) is reached or until the child node exists within the current range of *R_b_* away from the parent node.

To minimize overlap among adjacent sub-trees, each root node selects the farthest leaf node as the next root. The preceding steps are taken to optimize a network from local to global.

The proposed algorithm has four phases. (1) The root node constructs a sub-tree and selects the next root node; (2) The root node calculates the dive depth of each leaf node; (3) The depth of each leaf node is adjusted, while the next root begins a new round of calculation; (4) The depths of certain omissive nodes are adjusted randomly. Each phase is described in detail.

The status flag bit (used to mark the node in terms of whether depth adjustment is achieved) of all the sensor nodes is initialized to 0, and the sink node begins to perform the following steps as the first root node.

#### 4.3.1. Constructing a Sub-Tree and Selecting the Next Root Node

Initially, the root broadcasts inquiry packets with radius *R_b_* and the nodes within the range of *R_b_* receive the inquiry packets. Then, the state flag is checked. If the state flag is 1, then this message is disregarded; otherwise, return the node number and coordinates. *R_b_* is determined through Equation (6):

(6) $$R_{b}=\{\begin{array}{ccc} \;R_{c}\;\; & \text{if}\; & R_{c}<2\times R_{s}\\ 2\times R_{s}\; & \text{if}\; & R_{c}\geq 2R_{s} \end{array}$$

After waiting for time *T*, [2 × (*R_c_* /*P* + *T_d_*)], the definition of the symbols is related in Section 5.2, the root node receives the information of the number and the coordinates from the nodes within the range of *R_b_* with a state flag of 0. Then, the root node uses these nodes as leaf nodes to build a sub-tree set. If the number of total leaf nodes is 0 and *R_c_* > 2 × *R_s_*, then the root node gradually adjusts the broadcast radius according to Equation (7):

(7) $$R_{b}=2R_{s}\text{+}\;\frac{R_{c}-2R_{s}}{k}i\;(i=1,2,\ldots ,k)$$

where *k* is the adjustment level and *i* represents the *i*th adjustment.

Upon establishing a sub-tree set, the sink node calculates the distance between each two nodes within the sub-tree set. Assuming that all nodes are initially on the same horizontal plane, the depth of each node is 0. In this case, the distance between two nodes is the horizontal distance (*Dis_horizontal*), and the established table is the horizontal distance table (*Dis_horizontal_table*). After the table is established, the root node selects the farthest node away from the root node as the next root, which is called root 1. If the there are more than two leaf nodes, the root node selects the farthest node away from root 1 as root 2.

#### 4.3.2. Calculating the Depth of Each Node

The root node begins to calculate the dive depth of each node within the sub-tree cluster, and the root node is regarded as the first parent node.

First, the root node determines the nearest node away from the parent nodes in the sub-tree cluster as the child node, and then calculates node depth according to Equations (8) and (9). After calculation, the root node sets the state of this node for completion.

(8) $$\begin{array}{l} Dis\_vertical(parent,son)=\sqrt{{R_{b}}^{2}-Dis\_horizontal{\left(parent,son\right)}^{2}} \end{array}$$

(9) $$\begin{array}{l} Depth(son)=Dis\_vertical(parent,son)+Depth(parent) \end{array}$$

Equation (8) represents the relative vertical distance between the parent and child nodes, whereas Equation (9) represents the final depth of the leaf node.

**Figure 2:**
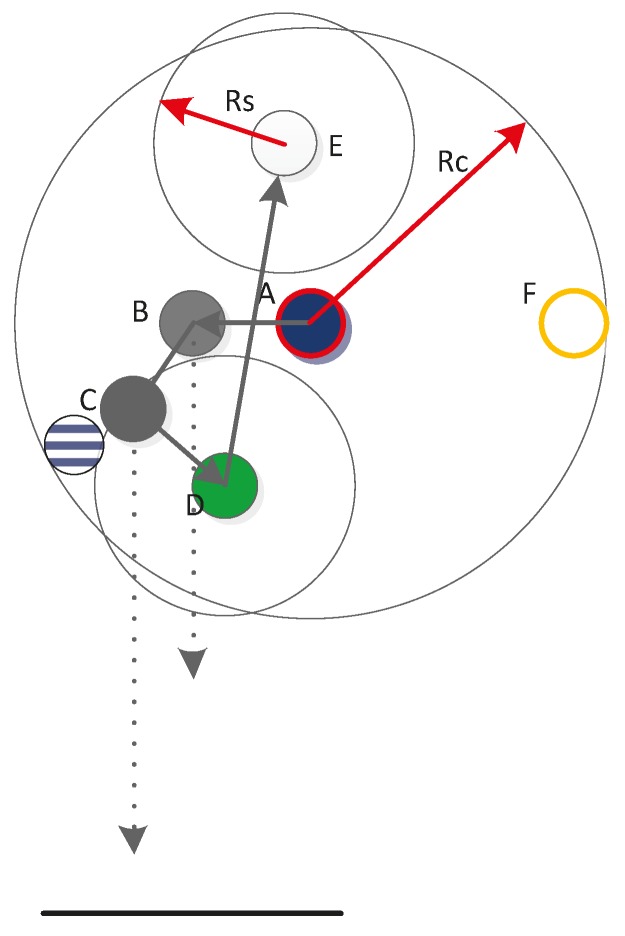
First example of node calculation.

**Figure 3:**
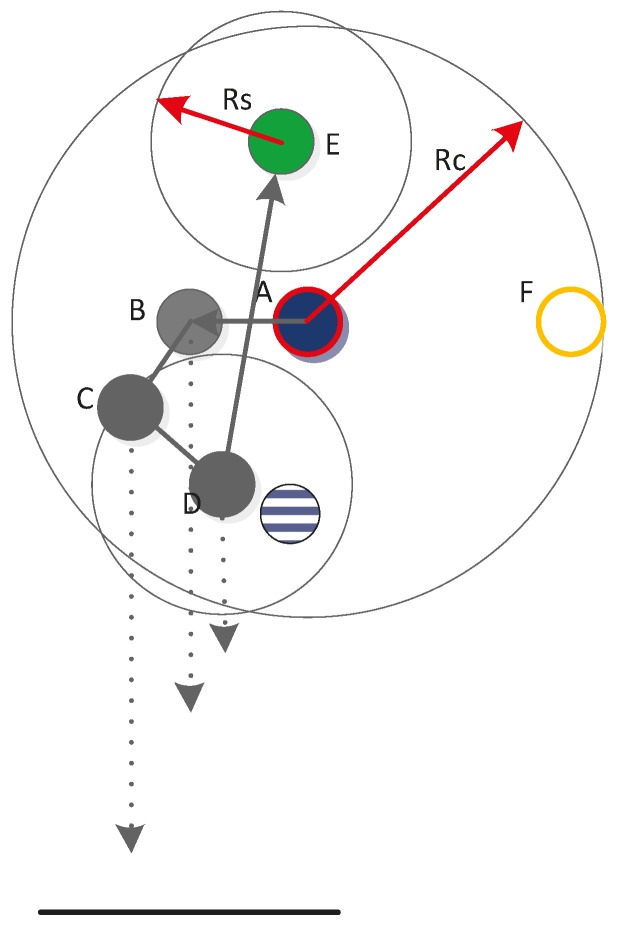
Second example of node calculation.

After calculation, the just-completed node is updated as the parent node, and the root node continues to regard the node in the sub-tree cluster that is nearest to the parent node as the child node. The root node then begins the next round of calculation. If *Dis_horizontal(parent*, *son) > R_b_* or *Depth(son)* is larger than the depth of the monitored area, then the parent node is set as the root node and the dive depth of the child node is recalculated. If the leaf node is the next root node, then its parent node is also set as the root node; meanwhile, the maximum depth is limited to *R_s_*. As shown in Figure 2 and Figure 3, the circle with the red side is the root node, the circle with the yellow side is the next root node, the pure blue circle represents the parent node, the pure green circle is the child node, the pure grey circle denotes that node depth has been calculated, and the black arrows indicate the calculation order in the sub-tree. As shown in Figure 2, following the normal sequence, when the depth of node *D* is calculated, the parent node should be node *C* (blue and white circles in Figure 2), but the result obtained is *Depth(D) > Depth(water)*. Thus, the parent node of node *D* is updated as the root node and the depth of node *D* is recalculated, as shown in Figure 2. Next, the root node calculates the depth of node *E*, and node *D* becomes the parent node, as indicated in the normal order (blue and white circles in Figure 3). However, *Dis_horizontal (D*, *E) > R_b_*; thus, the parent node is updated as the root node, as shown in Figure 3. When the depth of node *F* is calculated, its parent node is also updated as the root node.

#### 4.3.3. Broadcast Configuration Messages and Depth Adjustment

After calculating the depths of all the leaf nodes, the root node begins to broadcast a configuration message, which includes the node number and the corresponding dive depth. If the leaf node is the next root, one or two more bit data should be added to indicate this information. When the leaf node receives the message, this node sets its own state flag to 1, and then starts to adjust its depth. If a node is the root, then this node starts running the algorithm from Step 1 to build its sub-tree cluster and calculate the dive depth of its leaf nodes. After determining the depth of all the leaf nodes, the root node starts to adjust its own depth.

#### 4.3.4. Random Adjustment

Since sensor nodes are randomly and uniformly distributed on the water surface initially, some nodes may have not any neighbor nodes in the communication range, these nodes are isolated, and the preceding steps cannot involve these nodes. To cover more space and find possible neighbor nodes in a simple and feasible way, these nodes adjust the depth randomly. Algorithm 1 shows the pseudo-code of the algorithm.
**Algorithm 1** Pseudo-Code Executed on the Root Node1. Broadcast *inquiry_message*2. Receive *respond_message*3. Set up *sub-tree* cluster and select next root4. **While** the *sub-tree* cluster is not empty5. Calculate each leaf node depth6. **End**7. Broadcast *configuration_message*8. Adjust own depth


## 5. Algorithm Analysis

The message and run-time complexity of CTDA are evaluated in this section.

### 5.1. Message Complexity

The total number of sent and received messages is considered to determine the message complexity of CTDA.

If node *i* is the root node, then this node will broadcast inquiry messages with radius *R_b_* The nodes within the range of *R_b_* receive the inquiry packets and check their own state flag. If the state flag is 1, then the message is disregarded; otherwise, the nodes return their original node number and coordinates. After waiting for *T*, the root node builds a sub-tree using the nodes that answer the inquiry message. If the total number of leaf nodes is 0, then the value of *R_b_* is gradually adjusted until *R_b_* reaches *R_c_* or the total number of leaf nodes is no longer 0. Given that the max adjustment level is *k*, the root node can broadcast *k* + 1 times at most. Assuming *M_j_(M_j_* ≤ *n)* nodes exist within the last broadcast radius range, the number of nodes with a state flag of 0 is *N_j_*; thus, the root node in each sub-tree receives *N_j_* pieces of response packets, and then broadcasts *N_j_* pieces of configuration messages. Assuming that the total number of root nodes in the network is *c*, where *c* ≥ 1, the total number of packets sent by all the root nodes in the network is

(10) $$total\_root\_send=(1+k)c+\sum_{j=1}^{c}N_{j}$$

The total number of packets received by the root nodes is

(11) $$total\_root\_receive=\sum_{j=1}^{c}N_{j}$$

The total number of packets sent and received by all the regular nodes in the network is

(12) $$total\_ordinary\_send=\sum_{j=1}^{c}N_{j}$$

(13) $$total\_ordinary\_receive=\sum_{j=1}^{c}N_{j}+\sum_{j=1}^{c}M_{j}$$

Hence, the total number of sent packets in the network is

(14) $$(1+k)c+\sum_{j=1}^{c}N_{j}\text{+}\sum_{j=1}^{c}N_{j}\leq (1+k)c+2n$$

with the complexity is *O*(*n*). The total number of received packets is

(15) $$\sum_{j=1}^{c}N_{j}+\sum_{j=1}^{c}N_{j}+\sum_{j=1}^{c}M_{j}\leq 2n+\sum_{j=1}^{c}M_{j}\leq 2n+cn$$

with the complexity is *O*(*cn*).

### 5.2. Run-Time Complexity

The run-time complexity required to complete network deployment is evaluated in this section. The related symbols used are defined in Table 1.

In CTDA, the time required to complete network deployment depends on the ending time of the last connected sub-tree. When *R_b_* is equal to *R_c_*, the time that the root node spends to construct a sub-tree is the longest. In this case, the root node broadcasts (1 + *k*) times; thus, the maximum time required to build a sub-tree is (1 + *k*) × 2 × (*R_c_/P + Td*). The root node then calculates the depth of each leaf node and broadcasts the configuration messages, which takes time (*E* + *R_c_/P* + *Td*). Therefore, the maximum time for the leaf nodes in the last sub-tree to receive the configuration packet completely is (*c* − 1) × [(1 + *k*) × 2 × *R_c_/P* + *Td* + (*E* + *R_c_/P* + *Td*)]. After receiving the configuration message, the leaf node begins to adjust its depth. The worst-case scenario is that some nodes should be moved to the bottom and the time is *D/V*. Hence, the maximum time required to complete network deployment is (*c_1_*) × [(1 + *k*) × 2 × *R_c_/P + Td +* (*E + R_c_/P + Td*)] *+ D/V*, and the run-time complexity of CTDA is *O* (*R_c_/P + T_d_ + E + D/V*).

**Table 1:** Definition of symbols in run-time complexity analysis.

Total depth	*D*
Speed of sound	*P*
Transmission delay	*T_d_*
Vertical node speed	*V*
Number of connected sub-trees	*C*
Algorithm execution time	*E*

UWSNs typically adopt acoustic communication. The average propagation velocity of sound in water is 1500 m/s, and the time required for acoustic wave to spread to 1000 m is 0.67 s. Given the limitation of current technology, the speed of the sensor nodes in the vertical direction is generally slow; for example, the movement speed of the sensor node described in [30] is 2.4 m/min. Therefore, the time required to complete network deployment primarily depends on the speed of the sensor node and the depth of the monitored area.

## 6. Simulation Evaluation

This section describes the simulation setup, the simulation performance, and the related analysis.

### 6.1. Simulation Setup

To analyze the effectiveness of CTDA, simulations and comparisons are performed on the network coverage, connectivity, and deployment energy consumption of CTDA, SDDA, RAND, and CDA. The reason why SDDA and CDA are chosen for comparison is that both of them are typical depth adjustment algorithms, and RAND is chosen as the blank comparison. In addition, the causes of the results are analyzed. In fact, Senel *et al.* [22] also choose SDDA as comparison with CDA to illustrate the performance of CDA. Therefore, the simulation setup is set the same with SDDA: The volume of the deployment area is 35.7 m *×* 35.7 m *×* 53.9 m, the sensing radius of the node is 10 m, the communication radius of the node is 17.9 m. The difference between the present study and [21] is that the sink nodes are not located at the center of the water surface; instead, they are randomly deployed on the water along with the sensor nodes, which is more practical. To calculate network coverage, the monitored area is divided into 35 *×* 35 *×* 53 small cubic lattices. The volume of each cubic lattice is 1 m *×* 1 m *×* 1 m. A probe point is placed at the apex of each cubic lattice, since sensor nodes are randomly and uniformly distributed on the water surface initially, to eliminate the influences of the experiment randomness, the simulation result is the mean of 100 experiments.

### 6.2. Simulations

#### 6.2.1. Coverage

**Figure 4:**
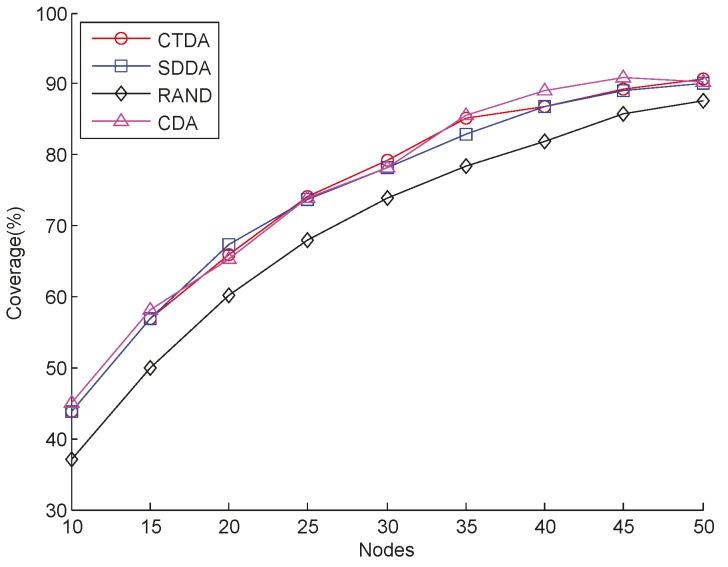
Coverage comparison with varying numbers of nodes.

First, *R_c_* is set to 17.9 m and *R_b_* to 10 m. Then, the coverage performance of CTDA is assessed and compared with those of SDDA, RAND, and CDA under different nodes. The results are presented in Figure 4. Evidently, coverage increases as the number of nodes increases for all the aforementioned algorithms. Using the same number of nodes, the coverage values of CTDA, SDDA, and CDA are close to one another and are higher than that of RAND. This result is attributed to the fact that the cluster head in SDDA assigns a group number based on whether a coverage overlap exists between two nodes. If an overlap exists, then the two nodes are assigned with different group numbers. After adjustment, a coverage overlap may still occur between two neighboring nodes, which, in this case, indicates that the depth of the nodes should be continuously adjusted to further reduce overlap and improve overall coverage. In CTDA, the root node selects the leaf node, which has the greatest coverage overlap with its parent node as a child node. Adjusting the distance between the two nodes minimizes overlap under the premise of maintaining connectivity to maximize coverage. CDA initially constructs the connected backbone, and then uses the dominating node on the backbone to calculate the depth of the dominated node of the backbone iteratively. The process can be described as follows. First, some possible depths of a dominated node are determined by the dominating node. In addition, the corresponding coverage that overlaps between the dominated node and the other deployed nodes at different depths of the dominated node is also calculated. Finally, the depth that corresponds to the minimum coverage overlap is selected as the final depth of the dominated node. In RAND, the depths of the nodes are randomly adjusted, which may expand the coverage overlap area between nodes.

**Figure 5:**
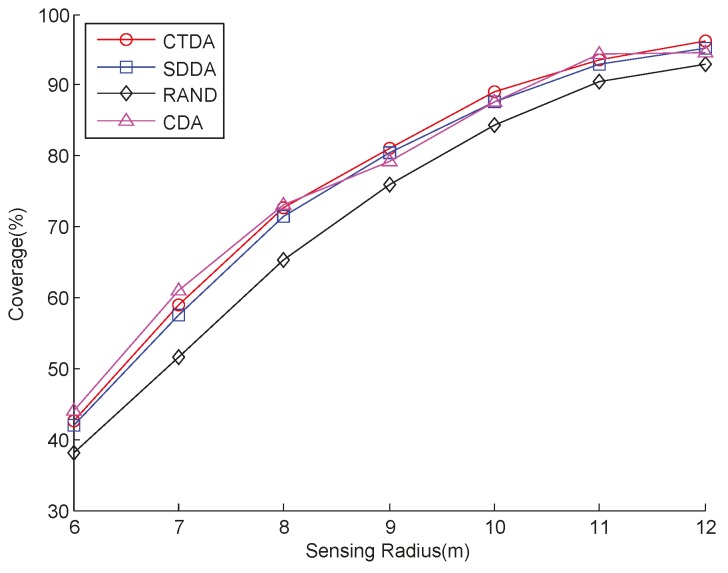
Coverage comparison with varying sensing ranges.

An experiment is also conducted by setting *R_c_* to 17.9 m and the number of nodes to 43, and by increasing *R_s_*. The coverage performances of CTDA, SDDA, RAND, and CDA are shown in Figure 5. The performances of these four algorithms are similar to those shown in Figure 4.

#### 6.2.2. Connectivity

Two different experiments are conducted to access connectivity performance by varying both the number of nodes and *R_c_*.

**Figure 6:**
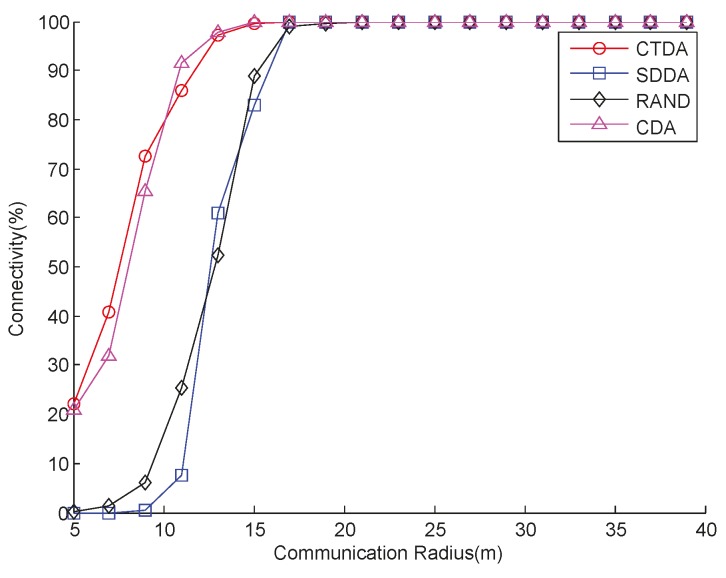
Connectivity with varying communication radii.

Figure 6 shows the comparison of the connectivity that varies with the *R_c_* of the four algorithms when the number of nodes is 43 and *R_s_* is 10 m. As shown in Figure 6, when *R_c_* is less than 20 m under the same value, SDDA performance is slightly better than that of RAND. CTDA achieves a better result than SDDA and RAND. CDA performs closely with CTDA. This result can be attributed to the fact that SDDA mainly focuses on maximizing network coverage, and that some nodes that are originally connected lose contact with the sink node after depth is adjusted to improve network connectivity. The node does not only continuously adjust depth but also increase energy consumption and possibly decrease coverage. In CTDA, building a connected tree ensures that the node that is initially connected still maintains connectivity after adjusting depth as much as possible. In CDA, the network initially builds a connectivity link, and then, as the leader, the node on the link calculates the depth of the surrounding nodes. When *R_c_* is greater than 20 m, the three algorithms can all achieve full connectivity [32].

**Figure 7:**
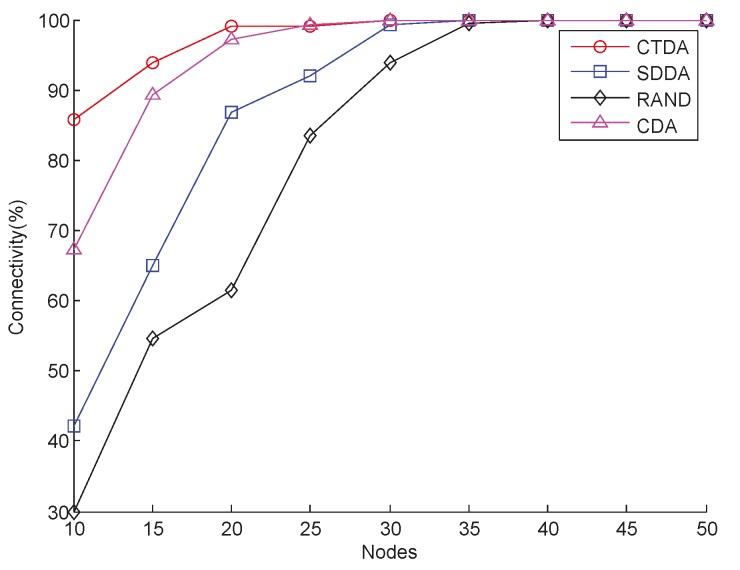
Connectivity with varying numbers of nodes.

With varying numbers of nodes, *R_s_* is 10 m and *R_c_* is 17.9 m, connectivity can also be guaranteed, as shown in Figure 7. Similar to Figure 6, CTDA always maintains high network connectivity, which is above 80% and higher than those of RAND and SDDA. However, the connectivity of CTDA is slightly higher than that of CDA.

#### 6.2.3. Deployment Energy Consumption

Network deployment energy consumption consists of communication energy and node movement energy consumptions. In this study, the total number of packets that the entire network sends and receives is employed, and the sum of the moved distance of all the nodes indicates the corresponding evaluation.

The main factors that affect the total number of received and sent packets are the number of nodes and the value of *R_c_*. Therefore, the total number of packets is counted under two different configurations. First, the number of nodes is 43 and the value of *R_c_* changes. Second, the value of *R_c_* is 17.9 m and the number of nodes varies. In CTDA, if a node is selected as the next root node, then the current root node requires additional 1–2 bit data in the configuration packets to indicate such information. For statistical convenience, all the packets are assumed to have the same size.

As shown in Table 2 and Table 3, the number of sent and received packets for SDDA, CTDA, and CDA increases with the number of nodes. Under the same number of nodes and *R_c_* value, the number of sent and received packets of the CTDA network is less than those of SDDA and CDA, particularly the total sent packets. This result is attributed to the fact that SDDA uses the *active* communication mode during clustering, and thus, nodes should be further adjusted to send messages actively. In CDA, nodes are required to broadcast messages actively when building the connectivity link and joining leader nodes. However, all nodes adopt the silent mode in CTDA, except the root node, and nodes send messages only after receiving an inquiry message, which considerably reduces network communication traffic.

**Table 2:** Total messages with various communication ranges.

	Total Sent Messages			Total Received Messages		
*R_c_*(*m*)	SDDA	CDA	CTDA	SDDA	CDA	CTDA
10	411	156	66	1171	969	602
15	419	172	92	2237	2071	1808
20	461	172	90	3187	2995	2378
25	435	174	90	4107	3642	2924
30	445	172	89	4393	3992	3002

**Table 3:** Total messages with various numbers of nodes.

Numbers of Nodes	Total Sent Messages			Total Received Messages		
	SDDA	CDA	CTDA	SDDA	CDA	CTDA
10	74	32	21	186	144	120
15	117	58	33	477	438	312
20	162	78	41	656	581	532
25	221	98	44	781	701	674
30	280	120	65	1380	1256	1192
35	337	142	74	2061	1743	1622
40	404	158	85	2612	2423	2336
45	593	182	95	3033	2746	2602
50	620	206	104	4392	4042	3086

**Figure 8:**
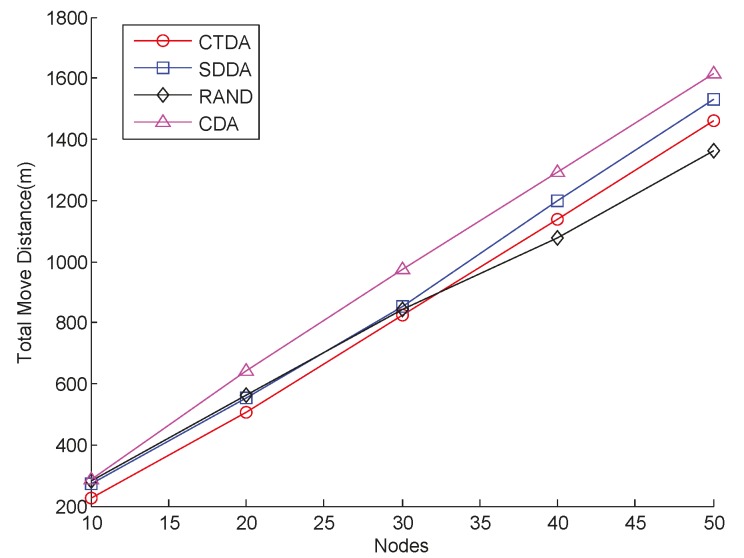
Comparison of total moved distance at varying numbers of nodes.

A 3D coverage is obtained by adjusting the depth of nodes. Obviously, the longer the distance moved by nodes, the greater the amount of energy consumed. Experiments are conducted using different numbers of nodes to determine the total distance moved by nodes and compared the values obtained in SDDA, RAND, and CDA. The result is provided in Figure 8. Evidently, the total distance moved in the three algorithms increases with the number of nodes. When the number of nodes is less than 30, the total distance of CTDA is the smallest under the same number of nodes; when the number of nodes is greater than 30, the total distance moved in CTDA is greater than that in RAND, but is always smaller than those in SDDA and CDA. The reason for this result is that when there are only few nodes in CTDA, the constructed tree is relatively small, and only nodes that move a short distance will reach the calculated position. As the number of nodes increases, the sub-tree gradually grows taller; thus, the node should move at a longer distance, which increases the total distance. For CDA, the location of each node is selected from above or below the deployed nodes; thus, the span of the candidate positions is extensive. If too many nodes are deployed below the deployed nodes, then the moving distance of the nodes increases substantially. In SDDA, some nodes require continuous depth adjustment after the first round to reduce overlap or improve connectivity. When the number of nodes increases, total travel distance also increases.

**Figure 9:**
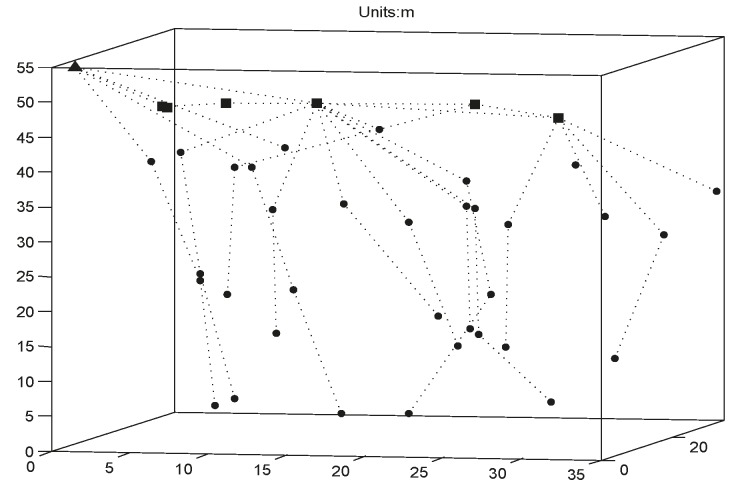
Node distribution map.

Figure 9 shows the node distribution map after CTDA is run once when the number of nodes is 43, *R_s_* is 10 m, and *R_c_* is 17.9 m. In the figure, the triangle denotes the sink node, the square represents the root node of a tree, the circle stands for the ordinary node, and the dashed line indicates the existence of a parent-child relationship between two nodes.

#### 6.2.4. Simulation under a Sparse Environment

In practical applications, sensor nodes are generally expensive, and acoustic signal propagation is significantly longer than those used in the previous section. Compared with terrestrial sensor networks, underwater sensor networks are commonly sparse networks. In this section, the performances of these algorithms under a sparse network environment are discussed.

The regular working communication range of the underwater sensor node described in [30] is 100 m and reaches a maximum of 400 m. Hence, the communication range is reset from 100 m to 400 m, volume is increased to 500 m × 500 m × 500 m, and the number of nodes is set to 60. Given that the sensing range of a node is determined by the characteristics of the perception sensor nodes and generally does not change significantly, the range is still set to 10 m. Moreover, the total volume is (500 m)^3^ = 1.25 × 10^8^ m^3^, the maximum volume covered by the 60 sensor nodes is 60 × (3/4) × 3.14 × 103 = 1.413 × 10^5^ m^3^, which is considerably smaller than the total volume. Therefore, coverage is not discussed in this section.

**Figure 10:**
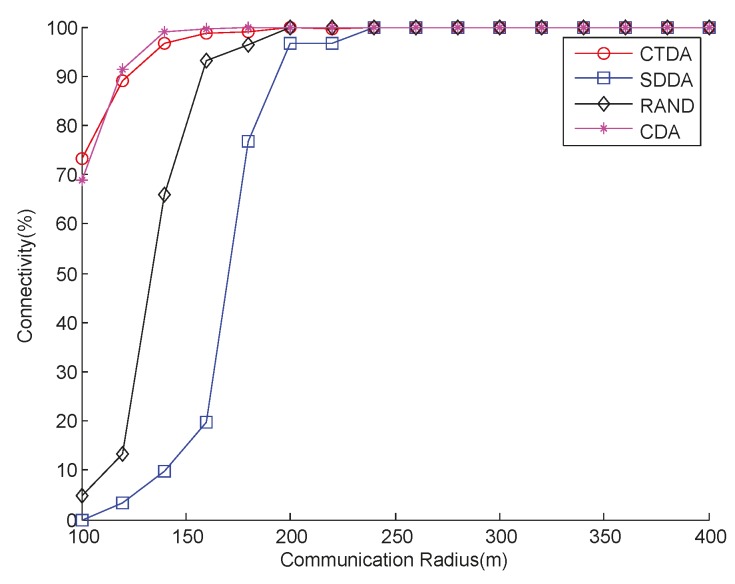
Connectivity with varying communication radii under a sparse environment.

Figure 10 presents the comparison of the four algorithms in terms of connectivity under a sparse environment. As shown in the figure, the connectivity of CTDA always maintains a high position. This position further proves the effectiveness of CTDA. In Figure 10, SDDA performs the worst because as the network becomes sparse, the number of nodes with a coverage overlap with neighboring nodes is reduced, an increasing number of nodes are assigned with the same group number, and the distance between two groups widens. If the communication radius is smaller than the inner-group distance, then the connection between two groups fails, and the whole network becomes disconnected in the worst-case scenario. Therefore, to improve connectivity, node depth requires further adjustment, which increases energy consumption.

**Figure 11:**
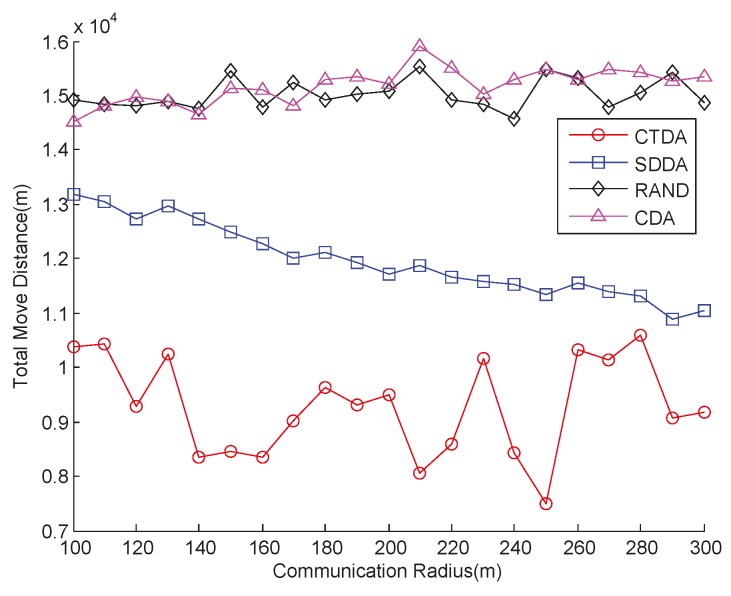
Comparison of distances moved with varying communication radii under a sparse environment.

Meanwhile, the total distances moved in the four algorithms under a sparse environment are compared. The results are presented in Figure 11, which indicates that the total distance moved in RAND and CDA is the largest, with fluctuations of approximately 1.5 × 10^4^ m, which is roughly half the water depth multiplied by the number of sensor nodes. The total distance moved in SDDA is less than those in RAND and CDA, and slowly decreases as communication radius increases; the total distance moved in CTDA is the shortest. Given that a node moves randomly in RAND, many unnecessary movements occur. However, given the large communication radius of CDA in a sparse environment, this algorithm widens the span of node adjustment and increases moving distance. In SDDA, when the communication radius of a node increases, the number of cluster members also increases, which may raise the group number, reduce interlayer distance, and shorten the moving distance of some nodes. In CTDA, the distance moved by nodes can be reduced effectively by gradually adjusting the distance between the root and child nodes within a tree. A high adjustment level indicates that good performance is achieved but the communication energy cost of the root node will be high.

#### 6.2.5. Simulation under a Dynamic Network Environment

In some underwater sensor network applications, the position of sensor nodes is not fixed after deployment. In addition, the nodes are affected by water current, which enables them to move randomly in the horizontal direction. However, the location changes of nodes may probably change network connectivity. Hence, assessing the connectivity performance of the four algorithms is necessary by considering the influences of water current.

The mobility model presented in [33] is used to describe the movement of nodes in water. In this case, the position of a node changes with the changing water environment. Therefore, the connectivity performance of the three algorithms is determined at different periods.

Upon completion of network deployment, the sink node with sensor nodes moves horizontally under the influence of water, and network connectivity is calculated every 60 s. The speed of a node in the *x* and *y* directions is calculated by Equation (16):

(16) $$\{\begin{array}{c} V_{x}=k_{1}\lambda v\text{sin}(k_{2}y)\text{cos}(k_{3}y)+k_{1}\lambda \text{cos}(2k_{1}t)+k_{4}\\ V_{y}=-\lambda v\text{cos}(k_{2}x)\text{sin}(k_{3}y)+k_{5} \end{array}$$

where *k_1_*, *k_2_*, *k_3_*, and *v* are the variables related to the environment; and *k_4_*, *k_5_* are random variables, whose values are as follows: *k_1_*, *k_2_ ~ N* (π, 0.1π), *k_3_ ~ N* (2π, 0.2π), *k_4_*, *k_5_ ~ N* (1, 0.1), *λ* ~ N (3, 0.3), *v ~ N* (0, 0.1). The location of a node is updated by Equation (17):

(17) Loc(i)=Loc(i−1) + Tv(i)

where *Loc*(*i*) represents the node location at time *i*; *Loc* (*i* 1) is the location at time *i −* 1; and *T* is the interval time for updating the location, which is determined by Equation (16).

**Figure 12:**
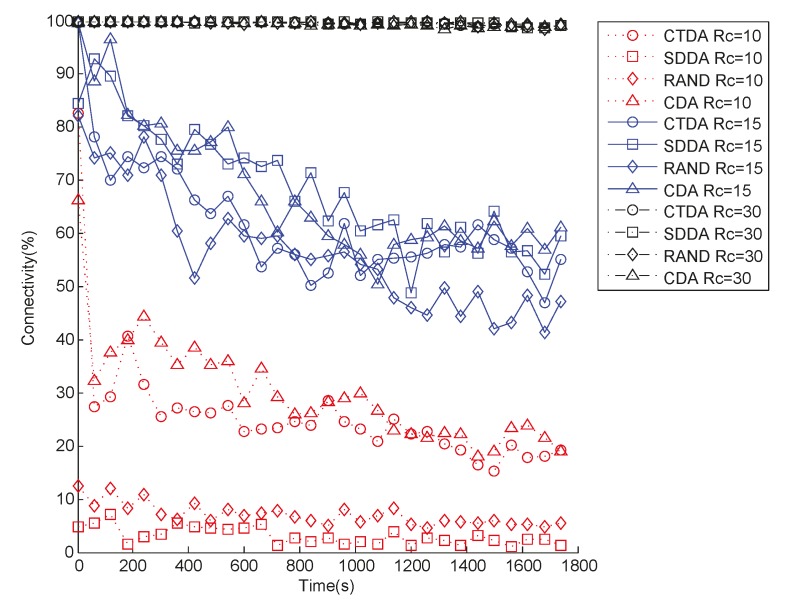
Connectivity varies with time under a dynamic environment.

Figure 12 shows the comparison chart when the initial deployment area is 35.7 m × 35.7 m × 53.9 m and the number of nodes is 43. Evidently, when the value of *R_c_* varies, the connectivity of the four algorithms decreases with time at different rates. When *R_c_* is 10 m, network connectivity declines the fastest, whereas network connectivity declines the slowest when *R_c_* is 30 m. This result is attributed to the fact that when *R_c_* is small, minimal changes in position cause a rapid decline in connectivity. When *R_c_* is 10 m, the connectivity of CTDA decreases the fastest in the initial period. To maximize network coverage when *R_c_* is less than 2 × *R_s_*, the distance between the parent and child nodes is set as equal to *R_c_*. Thus, the connectivity between the root and child nodes is vulnerable. The distance can be reduced by sacrificing certain network coverage to improve connectivity quality among nodes. When *R_c_* gradually increases from 10 m to 30 m, the effect of position change on network connectivity is decelerated.

## 7. Conclusions

Node deployment is one of the fundamental issues in UWSNs. A well-designed deployment algorithm can promote underwater monitoring quality or reduce network construction cost, among other aspects. This study presents a distributed deployment algorithm based on connected tree (*i.e.*, CTDA), where the sink node begins to build a connected tree as the first root node. Finally, a network is organized as a forest that comprises many connected sub-trees. Each root node adjusts the depth of its leaf nodes based on the coverage overlap between two nodes to minimize such overlaps and improve coverage. By hierarchically adjusting the distance between the parent and child nodes, the distance moved by a node is shortened. Moreover, the silent mode is adopted to reduce communication energy. CTDA is compared with RAND and SDDA in terms of network coverage, network connectivity, and deployment energy consumption under different node sensing radii, communication radii, and numbers of nodes. The simulation results show that CTDA maintains high network connectivity under different communication radii and numbers of nodes. Meanwhile, CTDA achieves network coverage as high as that of SDDA under different sensing radii and numbers of nodes. However, the deployment energy consumption of CTDA is less than that of SDDA. The simulation of a sparse network shows that the connectivity and deployment energy consumption performances of CTDA are significantly better than those of SDDA. Meanwhile, the connectivity and coverage performances of CTDA and CDA, which is similar to CTDA, are nearly the same. However, the energy consumption of CTDA is less than that of CDA, particularly in a sparse environment.
